# Measurement of saccharifying cellulase

**DOI:** 10.1186/1754-6834-2-21

**Published:** 2009-09-01

**Authors:** Douglas E Eveleigh, Mary Mandels, Raymond Andreotti, Charles Roche

**Affiliations:** 1 US Army Natick Development Center, Natick, Massachusetts 01760, USA

## Abstract

This article sets forth a simple cellulase assay procedure. Cellulose is variable in nature, insoluble and resistant to enzymatic attack. As a result there have been a bevy of bewildering cellulase assays published that yielded irrational results. Certain protocols focused on the rapidity of the assay while ignoring that only the most readily susceptible cellulose regions were being hydrolyzed. Other assays simplified the system by using modified soluble substrates and yielded results that bore no relationship to the real world hydrolysis of insoluble cellulose. In this study Mandels, Andreotti and Roche utilized a common substrate, Whatman filter paper. Hydrolysis of a 50 mg sample of the paper was followed to roughly 4% degradation, which circumvented the problems of attack of only the most susceptible zones. This common hydrolysis target range also resulted in some balance with regard to the interaction of the several cellulase components. The method was subsequently widely adopted.

Douglas E Eveleigh

## Introduction

As we move from laboratory research by microbiologists and biochemists to pilot plant and development studies by chemical engineers and industrialists, it is necessary to look at cellulose saccharification in a quantitative and economic manner. A major cost factor will be the cellulase enzymes. The engineer wishes a simple well-defined unit of cellulase on which he can put a dollar value based on capital and operating costs of fermentation, and from which he can predict sugar output in his reactor. This would appear to be a simple and easily satisfied requirement, but it is not. A bewildering array of substrates, enzyme actions, units, and activities have been used (Table 1). Part of the confusion is due to the tendency of workers to develop their own assays, and then modify them. As Matti Linko said in Finland [2] 'A biochemist would sooner use his colleagues' tooth brush than his assay procedure'. But most of the confusion is inherent in the multiplicity of both substrate and enzyme and the necessity of predicting the 40% to 50% conversion of a concentrated cellulose slurry from a reasonably short assay based on limited conversion of a much smaller quantity of substrate.

**Table 1:** Cellulase Assays

**Enzyme**	**Substrate**	**Product measured**
**Cellobiase**		
β Glucosidase	Cellobiose	Glucose
	Cellodextrins	
	Salicin	Salingenin
	p-Nitro β glucosidase	p Nitrophenol

**Endo β 1, 4 glucanase**		
C_x_	Carboxymethyl cellulose	Loss in viscosity
CMC'ase	Amorphous cellulose	Reducing sugar
	Walseth	
	*Sweco*	
	Cellodextrins	

**Exo β 1, 4 Glucanase**		
A Glucocellulase	Amorphous cellulose	Glucose (A)
	Walseth	
B Cellobiohydrolase	Crystalline cellulose	Cellobiose (B)
CBH	Avicel	
C_1_	Cellodextrins	

**Cellulase**		
C_1 _+ C_x_	Crystalline cellulose	Loss in weight
Avicellase	Avicel	Reducing sugar
Hydrocellulase	Hydrocellulose	Reduction in optical density (OD)
FP'ase	Filter paper	
	Cellodextrins	
	Cotton	

**Miscellaneous**		
Swelling factor	Cotton	Uptake of alkali
Filter paper cellulase	Filter paper	Maceration [1]
	Thread	Breaking strength
	Dyed cellulose	Release of dye

CMC = carboxymethyl cellulose; FP = filter paper.

**Figure 1:**
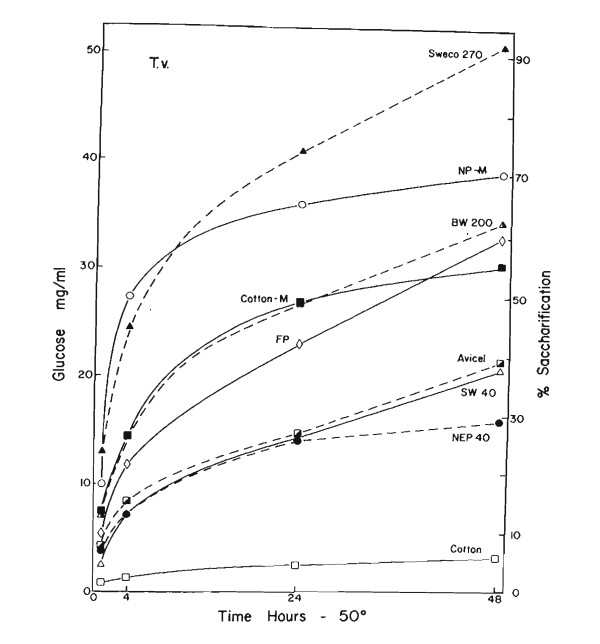
**Hydrolysis of insoluble cellulase by a complete cellulase from *Trichoderma viride *5% cellulase incubated at pH 4.8, 50°C with a filtrate of strain QM9414 grown on cellulose medium**. The enzyme preparation had 0.8 mg protein, 25 C_x _units, and 0.6 filter paper (FP) cellulose units/ml and a C_1 _activity of 2.8 mg of glucose/24 h. (white circle) Newspaper, Sweco ball milled; (black triangle) pure cellulose pulp, Sweco ball milled, 270 mesh; (white triangle) BW200, pure cellulose pulp, ball milled, Brown Co., Berlin, N.H.; (white diamond) Whatman No. 1 filter paper; (white square) ball milled absorbent cotton; (black square) Avicel pH 105, microcrystalline cellulose; (Δ-Δ) pure cellulose pulp, SW4O. Brown Co.; (black circle) hammer milled newsprint, NEP40, Brown Co.; (white square) absorbent cotton, fibrous.

Cellulose is deceptively simple chemically, a polymer consisting only of glucose linked only by β 1,4 bonds. But cellulose samples of different origin vary widely in chain length and the degree of interaction between the chains [3]. Furthermore, waste cellulose usually consists of only 40% to 60% cellulose with the balance consisting of hemicelluloses, lignins, and other materials. Many cellulase preparations also contain hemicellulases. If they are present, the hemicelluloses are rapidly hydrolyzed since they are much less recalcitrant to enzyme action than is cellulose. Amorphous cellulose is also rapidly hydrolyzed and then the rate of hydrolysis decreases greatly as the increasingly crystalline portions of the cellulose are attacked (Figure 1).

Cellulase is a complex of enzymes containing chiefly endo and exo β glucanases plus cellobiase. For complete hydrolysis of insoluble cellulose, synergistic action between the components is required. Since different cellulase preparations vary widely in the proportions of the different components, depending on source, growing conditions of the organism, and harvesting and handling procedures, the rate and extent of their hydrolysis of cellulose substrates also varies widely. Assay of purified components requires a variety of fairly complicated procedures that can be confusing to persons whose chief interest is in practical applications. In Finland (1975) the question arose 'What one substrate can be used to measure all the cellulase components?' Dr L G Petterson [4] opted for cellotetraose because it is acted on by all known members of the cellulase complex. Dr G Halliwell [5] decided on cotton because only a complete cellulase will hydrolyze it. So we had the choice of the most susceptible substrate or the most resistant, but both are unsatisfactory for a practical assay. Cellotetraose is not available commercially, but would have to be prepared by the investigator, a major research effort in its own right. Cotton is so slowly hydrolyzed that meaningful assays require 24 h. Finally, neither cotton nor cellotetraose is representative of a realistic substrate.

In the early studies on cellulase the available enzyme preparations would scarcely hydrolyze insoluble cellulose although they often broke down soluble derivatives such as carboxymethyl cellulose (CMC) readily. This was because they consisted chiefly of endo β glucanases (C_x_) and lacked the exo β glucanases (C_1_). This is still true for cellulase preparations derived from organisms like *Aspergillus niger *or from plant extracts. For such cellulases carboxymethyl cellulose is used as a substrate (Table 2).

**Table 2:** Endo β Glucanase (C_x_) Assay^a^

**Reference**	**Enzyme, ml**	**CMC, ml**	**CMC, % in assay**	**Time, min**	**1 C_x _units/ml Glucose mg/ml**	**1 C_x _units/ml Glucose total mg**	**International units** **C_x _units**
A^b ^[6]	1.0	9.0	0.5	60	0.4	4.0	0.37
B^c ^[7]	0.5	4.5	0.5	60	0.4	2.0	0.37
C^d ^[8]	0.5	0.5	0.5	30	0.5	0.5	0.185

^a^Substrate = carboxymethyl cellulose (CMC), degree of substitution 0.5. Temperature = 50 C. Product = reducing sugar as glucose. Buffer = 0.05 M citrate pH 4.8. Calculations: 1 IU = 1 μmol of glucose/min (0.18 mg/min). 1 mg glucose/0.18 × 60 = 0.0925 units (1 h) or 1 mg glucose/0.18 × 30 = 0.185 units (0.5 h).

^b^4.0 mg glucose × 0.0925/1.0 ml enzyme = 0.37 IU/C_x _unit.

^c^2.0 mg glucose × 0.0925/0.5 ml enzyme = 0.37 IU/C_x _unit.

^d^0.5 mg glucose × 0.185/0.5 ml enzyme = 0.185 IU/C_x _unit.

**Figure 2:**
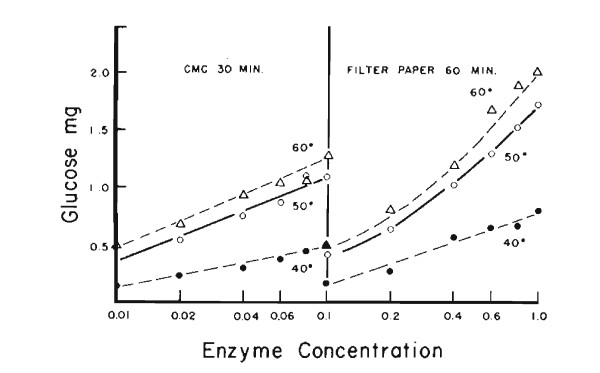
**Effect of enzyme concentration and of temperature on hydrolysis of carboxymethyl cellulose and filter paper by *Trichoderma viride *cellulase**. Culture filtrate of QM6a grown in cellulose, 12 C_x _units, 0.2 filter paper units/ml. Filter paper activity (1.0 ml) 1.74. Black circle, 40°C; white circle, 50°C; white triangle, 60°C.

Since the action on CMC is only linear to about 12% conversion due to interference by substituents (Figure 2), the units per ml were defined as the inverse of the dilution to give 0.4 or 0.5 mg/ml of reducing sugar as glucose with 0.5% carboxymethyl cellulose as the substrate in first a 1 h and later a 30 min assay. These units were of course arbitrary, but they are quantitative and can readily and preferably be converted to standard units according to the International Union of Biochemistry (that is, 1 unit equals 1 μmol of product per minute) (Table 2). A more serious deficiency is that the quantity of reducing sugars produced (and so the unit values) will be greatly affected by the particular sample of CMC used. The rate of hydrolysis is affected by both chain length and degree of substitution. Similar endo β 1,4 glucanase assays can be developed for other cellulose derivatives such as cellulose sulfate, but the unit values will not be directly comparable. Since CMC is soluble, it is readily hydrolyzed. *Trichoderma viride *cultures will yield 50 to 150 CMC units/ml with a specific activity of about 100 units/mg of protein. Other organisms such as *Pestalotiopsis westerdijkii *may yield as much as 400 CMC units/ml of culture filtrate.

**Figure 3:**
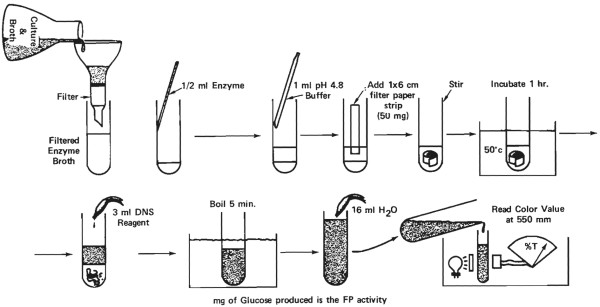
**Filter paper assay procedure**. Reagents: Whatman No. 1 filter paper cut into 1 × 6 cm strips (50 mg); buffer = 0.05 M Na citrate pH 4.8; glucose standards in buffer; dinitrosalicyclic acid (DNS). Reagent for reducing sugar [9]. Filter or centrifuge culture sample to remove solids. Dissolve enzyme powders at 1.0 to 5.0 mg/ml in buffer. Dilute enzyme solutions in buffer. Place 0.5 ml enzyme solution and 1.0 ml buffer in 18 mm test tube. Add a filter paper strip and mix on Vortex mixer to coil the paper in the solution. Incubate 1 h at 50°C. Add 3 ml DNS reagent to stop the reaction. Place tubes in boiling water for 5 min and determine the amount reducing sugar as glucose. Include a blank tube (without filter paper) to correct for any reducing sugar present in enzyme preparation. The mg of glucose produced in this test is the filter paper (FP) activity. The DNS reagent [9] measures reducing sugar non-specifically. When glucose is used as standard, values for cellobiose will be about 15% low and values for xylose about 15% high on a weight basis.

**Figure 4:**
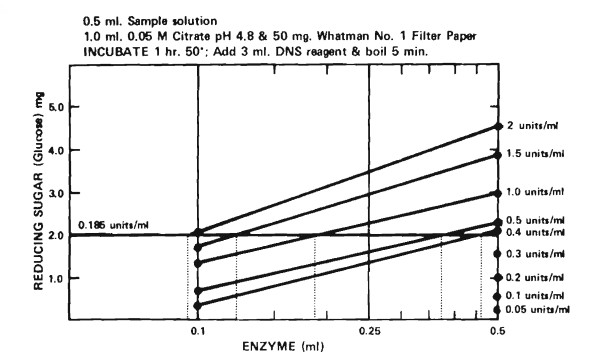
**Measurement of filter paper cellulase units per ml**. Follow procedure as outlined in Figure 3. If filter paper (FP) activity for 0.5 ml of enzyme is equal to or less than 2.0, units per ml equal FP activity × 0.185. If the FP activity is greater than 2.0, repeat using diluted enzyme and estimate the ml of enzyme required to give a FP activity of 2.0. Units per ml equals 0.185/ml of enzyme to give a FP activity of 2.0.

For a practical measurement of saccharifying cellulase measurement of endo β glucanase (or of any other single component) is unsatisfactory. For this reason the filter paper assay was introduced. It has the advantages of using a readily available and reproducible substrate that is neither too susceptible nor too resistant and that can be measured by unit area thus avoiding the tedium of weighing a solid or of trying to uniformly dispense a suspension of solids (Figure 3). The original filter paper (FP) activity referred to the amount of reducing sugar as glucose produced in the assay by 1 ml of enzyme. This was acceptable for monitoring fermentations or for comparing enzyme production by different strains but it was not quantitative (Table 3, Figure 4). For more quantitative work we used a unit based on 0.5 mg of glucose/ml analogous to the old C_x _unit [8].

**Table 3:** Filter Paper (FP) Activity and Units^a^

**International units/ml**	**Filter**	**Paper**	**Activity**		**ml enzyme for 2.0 mg glucose**
		
	**1.0 ml**	**0.5 ml**	**0.2 ml**	**0.1 ml**	
0.05	0.54	0.27	-	-	-
0.10	1.08	0.54	-	-	-
0.20	2.10	1.08	0.40	-	0.925
0.30	2.50	1.62	0.80	-	0.615
0.40	2.80	2.10	1.10	0.40	0.463
0.50	3.00	2.30	1.40	0.80	0.370
0.75	3.90	2.80	1.75	1.10	0.248
1.00	4.60	3.00	2.10	1.40	0.185
1.50	-	3.90	2.65	1.75	0.124
2.00	-	4.60	3.20	2.10	0.093

^a^Substrate: 50 mg Whatman No. 1 filter paper (6 cm^2^). Buffer: 1.0 ml, 0.05 M citrate pH 4.8. Enzyme: 1 ml assay, volume indicated + buffer to 1.0 ml FP activity × 0.0925 = IU/ml; 0.5 ml assay volume indicated + buffer to 0.5 ml FP activity × 0.185 IU/ml or if FP activity is greater than 2.0 IU/ml = 0.185/ml enzyme for 2.0 mg glucose. (2.0 mg glucose = 4% hydrolysis of 50 mg; the unit based on 0.5 mg of glucose from 1 ml of enzyme [8] is equal to 0.0463 IU.)

**Figure 5:**
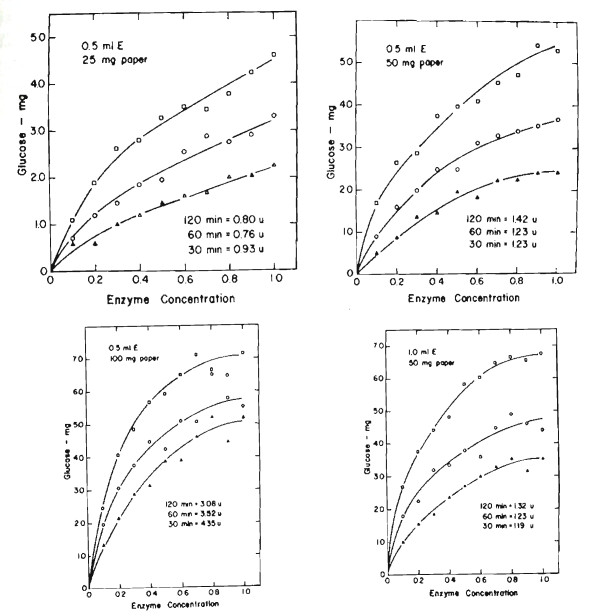
**Hydrolysis of filter paper by *Trichoderma viride *cellulase**. Effect of assay conditions, 0.5 or 1.0 ml enzyme + 1 ml buffer pH 4.8 + 25, 50, or 100 mg Whatman No. filter paper. Units = μmol of glucose/min based on dilution to give 2 mg of glucose. White triangle, 30 min incubation 50°C; white circle, 60 min incubation 50°C; white square, 120 min incubation 50°C.

Meantime in Peoria the assay was modified by increasing the cellulose to 100 mg and reducing the incubation time to 30 min but calculating the results back to a 60 min activity value [10]. We also modified the assay to use only 0.5 ml of enzyme [11,12]. At this point the literature and oral presentations were getting somewhat confused with the different assay procedures, loose references to FP activity as units, and calculating FP activities from shortened times or diluted enzyme preparations, giving apparently high but unreal values. A series of dilution curves (Figure 5) shows the effects of time, enzyme concentration, and cellulose concentration on the FP activity. It is evident that FP activity per unit of enzyme decreases with increasing enzyme concentration, that FP activity per unit of time decreases with increasing time of incubation, and that FP activity increases as cellulose concentration increases (Table 4).

**Table 4:** Effect of Conditions on Filter Paper Assay^a^

Filer paper mg		25	50	50	100
Enzyme ml		0.5	0.5	1.0	0.5
Enzyme protein mg		0.7	0.7	1.4	0.7
Activity mg glucose	30 min	2.29	2.43	3.52	5.18
	60 min	3.30	3.68	4.40	5.53
	120 min	4.60	5.30	6.75	7.16

Activity per h	30 min	4.58	4.86	7.04	10.36
	60 min	3.30	3.68	4.40	5.53
	120 min	2.30	2.65	3.38	3.58

Units/ml	30 min	0.93	1.23	1.19	4.35
	60 min	0.76	1.23	1.23	3.52
	120 min	0.80	1.42	1.32	3.08

Units/mg protein	30 min	0.66	0.88	0.85	3.11
	60 min	0.54	0.88	0.88	2.51
	120 min	0.57	1.01	0.94	2.61

^a^0.5 or 1.0 ml of enzyme (culture filtrate of QM9414 grown on cellulose) + 1 ml of pH 4.8 buffer + Strip of Whatman No. 1 filter paper. Units = μmol of glucose per min based on the dilution to give 2.0 mg glucose.

It was obviously time to start using a cellulase unit based on the international unit system but two problems arose. The first problem was what concentration of cellulose to use in the assay and the second problem was what extent of conversion was required for meaningful results. For a soluble substrate the answers are simple. Substrate level should be high enough that it does not limit the reaction, and the extent of conversion should be slight before depletion of the substrate or product inhibition affects the reaction rate. Because of the low bulk density of cellulose, concentrations greater than about *5% *become very thick and since cellulose is insoluble, the effective concentration is the available surface. Increasing the effective cellulose concentration by adding more of it or by milling the cellulose will increase the rate of the reaction and also make it more linear to a higher sugar value [10] but this increases the relative contribution of the enzymes acting on the more amorphous portions of the cellulose. We are more interested in the hydrolysis of the more crystalline and resistant portions by the whole cellulase complex. Filter paper, like other insoluble celluloses is a multiple substrate ranging from free ends and amorphous regions to crystalline fibers.

**Figure 6:**
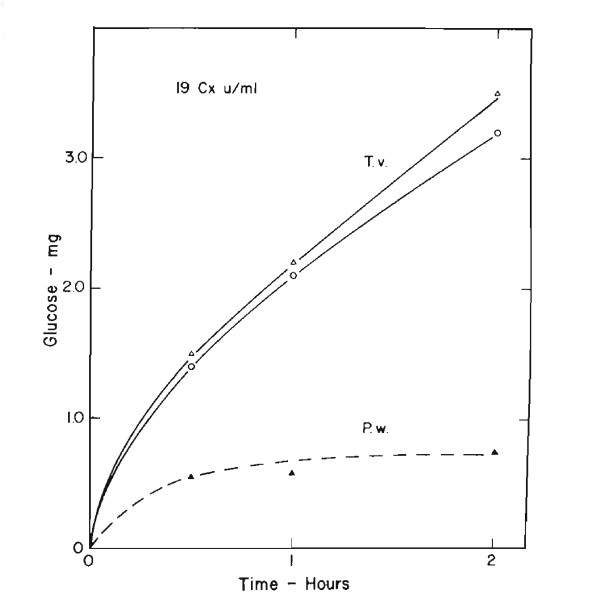
**Hydrolysis of filter paper by cellulase preparations from *Trichoderma viride *and *Pestalotiopsis westerdijkii *adjusted to equal activities on carboxymethyl cellulose**. Culture filtrates diluted to 19 C_x _units per ml. 0.5 ml enzyme + 1 ml pH 4.8 buffer + 50 mg paper. Incubated at 50°C. White triangle, T_v _QM9123 culture filtrate; white circle, T_v _QM9414 culture filtrate; black triangle, P_w _QM381 culture filtrate.

When culture filtrates of *P. westerdijkii *(which contain only endo β glucanases and β glucosidase) and *T. viride *(which is a complete cellulase) are diluted to equal activity on carboxymethyl cellulose, the initial hydrolysis by both preparations is rapid (Figure 6). The *Pestalotiopsis *cellulase, however, levels off at less than 1.5% hydrolysis by 30 min and this does not increase on longer incubation. The *Trichoderma *cellulase continues to hydrolyze the more resistant portions although at a slower rate. Recently, Dr Elwyn Reese [13] has produced cellulase filtrates from *Pestalotiopsis *with over 200 international endo β glucanase units per ml but they still have a FP activity of less than 0.8. So a meaningful cellulase assay should show a greater percentage conversion than this.

Moving to a more resistant substrate makes for a more rigorous assay but even cotton contains a little amorphous cellulose. For example, Stutzenberger [14] reported that the cellulase of *Thermomonospora curvata *contained C_1 _based on reducing sugar production from cotton. The international unit values looked pretty good because the assay time was only 10 min but the sugar level did not increase even after 30 h incubation. So the action appears to be on a limited (less than 1%) amorphous portion of the cotton. When we use cotton as a substrate, we incubate for 24 h and expect 5% to 10% conversion by *Trichoderma *preparations. Another proposal by Naylor [15] was to run the filter paper hydrolysis for a longer time and use the slope of the hydrolysis curve after 16 h. This is scientifically sound but time consuming and tedious if large numbers of assays are to be run.

Our solution to the problem has been to stay with the 50 mg of filter paper and use 0.5 ml of enzyme with 1 h incubation and to calculate international units as shown in Figures 3 and 4 from the dilution to give 2.0 mg of glucose (0.37 units/ml if the 0.5 ml assay is used). This cut-off value of 2.0 was chosen because the hydrolysis curves are fairly linear to beyond that level and because it represents 4% hydrolysis of the filter paper, well over the amount of sugar that could be expected from an incomplete cellulase. Higher unit values would result if the cutoff value were lower, if the assay time were decreased, or the cellulose concentration were increased.

**Figure 7:**
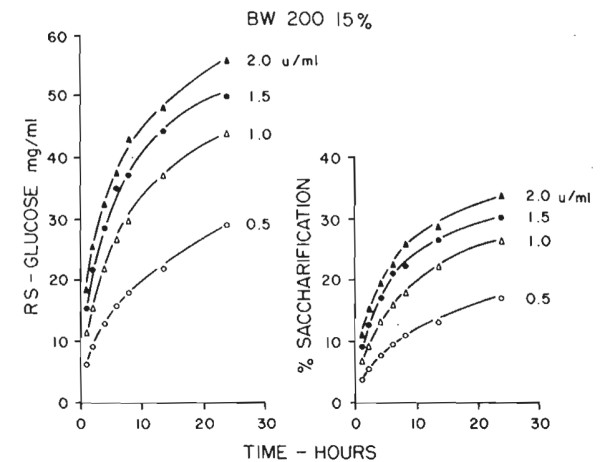
**Hydrolysis of milled cellulose pulp by *Trichoderma viride *cellulase**. 1 liter stirred tank reactor at 50°C, with 15% ball milled cellulose (BW200, Brown Co., Berlin, NH, USA), cellulase culture filtrate from QM9414, in 0.05 M citrate buffer. Percentage saccharification equals glucose mg/ml × 0.6.

*Trichoderma *cellulase fermentations as we are running them at Natick with the mutant strain QM9414 yield 1 to 2 units of cellulase/ml of culture broth for a specific activity of about 1 unit/mg of protein as determined by this assay. The amount of saccharification to be expected from such enzyme levels is shown (Figure 7) for pure milled cellulose.

In conclusion, the measurement of cellulase is complex and there is no absolute unit as can be measured for a single enzyme acting on a soluble substrate. The unit value will depend on the substrate chosen, its concentration, and the extent of conversion. The filter paper assay and unit value described here is not perfect but it is simple, reproducible, and quantitative and predicts enzyme action under practical saccharification conditions.
